# CCRR: a user-friendly platform for analyzing complex chromosomal rearrangements in tumors

**DOI:** 10.1093/bioinformatics/btaf386

**Published:** 2025-07-03

**Authors:** Jinjiang Liu, Kun Wang, Yawen Yuan, Guangchao Bao, Hang Ci, Mingqin Liu, Yunpan Lyu, Jingxin Tang, Jian Yang, Haoyang Cai

**Affiliations:** Center of Growth, Metabolism, and Aging, Key Laboratory of Bio-Resources and Eco-Environment, College of Life Sciences, Sichuan University, Chengdu, Sichuan 610064, China; Center of Growth, Metabolism, and Aging, Key Laboratory of Bio-Resources and Eco-Environment, College of Life Sciences, Sichuan University, Chengdu, Sichuan 610064, China; Center of Growth, Metabolism, and Aging, Key Laboratory of Bio-Resources and Eco-Environment, College of Life Sciences, Sichuan University, Chengdu, Sichuan 610064, China; Center of Growth, Metabolism, and Aging, Key Laboratory of Bio-Resources and Eco-Environment, College of Life Sciences, Sichuan University, Chengdu, Sichuan 610064, China; Center of Growth, Metabolism, and Aging, Key Laboratory of Bio-Resources and Eco-Environment, College of Life Sciences, Sichuan University, Chengdu, Sichuan 610064, China; Center of Growth, Metabolism, and Aging, Key Laboratory of Bio-Resources and Eco-Environment, College of Life Sciences, Sichuan University, Chengdu, Sichuan 610064, China; Center of Growth, Metabolism, and Aging, Key Laboratory of Bio-Resources and Eco-Environment, College of Life Sciences, Sichuan University, Chengdu, Sichuan 610064, China; Center of Growth, Metabolism, and Aging, Key Laboratory of Bio-Resources and Eco-Environment, College of Life Sciences, Sichuan University, Chengdu, Sichuan 610064, China; Center of Growth, Metabolism, and Aging, Key Laboratory of Bio-Resources and Eco-Environment, College of Life Sciences, Sichuan University, Chengdu, Sichuan 610064, China; Center of Growth, Metabolism, and Aging, Key Laboratory of Bio-Resources and Eco-Environment, College of Life Sciences, Sichuan University, Chengdu, Sichuan 610064, China

## Abstract

**Summary:**

Complex chromosomal rearrangements in tumors involve intricate genomic alterations that significantly affect gene function and contribute to cancer development. Identifying these events is crucial for cancer research but is often challenging due to the complexity and limitations of existing tools. We developed the Complex Chromosomal Rearrangements Resolver (CCRR), a comprehensive, reproducible, and user-friendly platform for analyzing complex rearrangements in tumors. CCRR integrates multiple SV and CNV detection tools within a Docker container environment, simplifying installation and configuration. It can be easily deployed, automating the execution and merging of results, providing high-confidence consensus SV and CNV calls, allowing researchers to efficiently analyze complex chromosomal rearrangements in tumors without extensive bioinformatics expertise. CCRR also includes a web server for one-click analysis and customized visualization.

**Availability and implementation:**

The CCRR platform is freely available at https://www.ccrr.life. Source code and executables can be accessed at https://github.com/laslk/CCRR. An archived version is available at Zenodo: https://doi.org/10.5281/zenodo.15386513.

## 1 Introduction

Somatic structural variations (SVs), including insertions and deletions, are genomic alterations that occur in specific cells or cell populations during an individual's development, resulting from errors in DNA repair, exposure to carcinogens, and other contributing factors. In contrast to simple structural variations, complex chromosomal rearrangements involve a greater number of breakpoints and more intricate alteration patterns, leading to widespread gene function loss or abnormal activation. Despite the high incidence of cell death during the formation of these chaotic genomes, this process may represent a powerful survival strategy for the genome under crisis (Pellestor, 2019). Identifying complex rearrangements in tumor genomes is crucial in cancer research (Yang *et al.* 2016, Baker *et al.* 2024). A standard WGS workflow for identifying somatic complex rearrangements typically begins with tumor/normal BAM file pairs, followed by the detection of structural variations and copy number variations (CNVs) to identify complex events (Cortés-Ciriano *et al.* 2022).

Accurately detecting SVs and CNVs is a major challenge in identifying complex rearrangements. Numerous bioinformatics pipelines and tools, such as Delly (Rausch *et al.* 2012), SvABA (Wala *et al.* 2018), Manta (Chen *et al.* 2016), and CNVkit (Talevich *et al.* 2016), have been developed. These tools utilize various detection algorithms to identify SV and CNV. However, the accuracy and consistency of SV and CNV identification are often low (Kosugi *et al.* 2019, Gabrielaite *et al.* 2021). Researchers frequently merge results from multiple tools, as several studies have shown that consensus results from different algorithms tend to have fewer false positives (The ICGC/TCGA Pan-Cancer Analysis of Whole Genomes Consortium 2020, Coutelier *et al.* 2022).

Another challenge arises from the independence of various complex rearrangement callers. Many tools are designed to detect only a single type of event, making it difficult to obtain a complete representation of complex rearrangements. For example, tools like ShatterSeek (Cortés-Ciriano *et al.* 2020) and CTLPScanner (Cai *et al.* 2014, Yang *et al.* 2016) are used to detect chromothripsis, AmpliconArchitect (Deshpande *et al.* 2019) to detect extrachromosomal DNA (ecDNA), and SeismicAmplification (Rosswog *et al.* 2021) to detect seismic amplification. These tools each require different input formats, dependencies, and operational methods, and some software receives limited maintenance, further complicating the analysis.

Here we introduce the Complex Chromosomal Rearrangements Resolver (CCRR), a user-friendly workflow for analyzing complex chromosomal rearrangements in tumors. CCRR provides a simple, versatile, flexible, and reproducible solution for detecting somatic complex rearrangement events in tumors. Based on Docker containerization technology (Merkel 2014), CCRR allows users to freely choose and install tools for detecting SV and CNV. It automatically installs the tools, configures dependencies, and uses a merge algorithm to obtain accurate consensus SVs and CNVs, enabling the execution of various complex rearrangement callers. To facilitate broader application, we also offer a CCRR web server. By uploading SV and CNV results from various tools or any custom SV and CNV data, users can achieve automatic one-click merging and obtain the analysis results of complex rearrangement events.

## 2 Software description

The CCRR workflow aims to detect complex somatic chromosomal rearrangements in tumors, allowing easy use on either Windows or Linux, high-performance servers or personal computers.

### 2.1 The CCRR workflow

The installation of CCRR begins with install.py. After the user specifies the needed tools and reference genome, this script automatically downloads the dependencies. The CCRR workflow is modular and controlled by the main program, ccrr.py. After installation, the Test mode runs selected tools with a small dataset to verify the installation. The Default mode executes the full analysis using a reference genome and normal/tumor BAM files. Users can also opt for the Fast mode, which quickly generates SV and CNV results, or the Custom mode for detailed customization.

**Figure 1.**
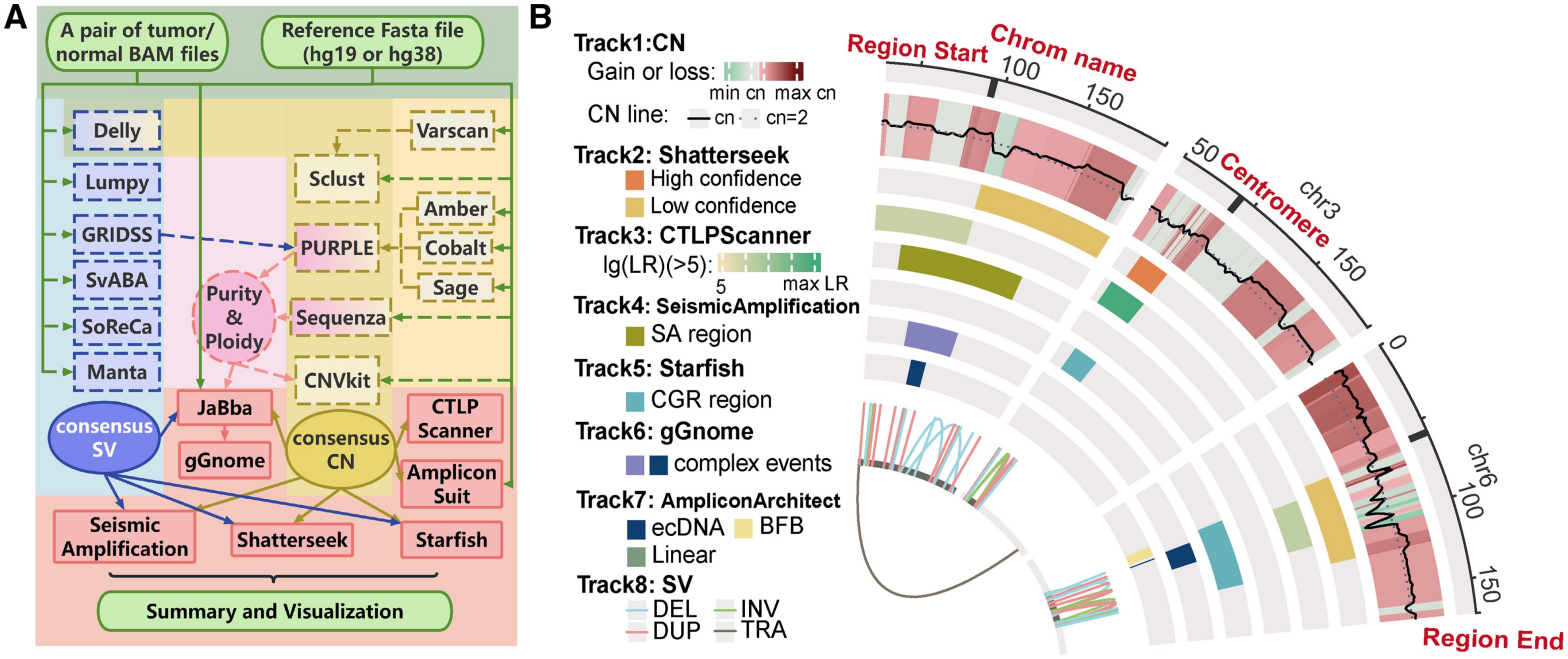
(A) Overview of the CCRR workflow. (B) Summary of CN, SV integration, and analysis results of various complex rearrangements by the CCRR workflow. The tracks, from outer to inner, display: (0) Chromosomes: shows the start and end points of chromosomal regions and the centromeres. (1) CN: Regional copy number gains or losses are indicated by distinct segments; a solid line represents a smoothed curve indicating actual copy numbers, with a straight line representing the default normal copy number state (CN = 2). (2) Shatterseek: Chromosomal shatter regions are indicated according to confidence level (criteria do not include statistical validation). (3) CTLPScanner: Areas identified as Chromothripsis-like Patterns based on the log likelihood ratio [lg(LR) ≥ 5]. (4) Seismic Amplification: Regions representing seismic amplification events. (5) Starfish: Marks complex genomic rearrangement areas. (6) gGnome: Indicates various complex event areas. (7) AmpliconArchitect: Different amplification types, including ecDNA, linear amplification, and BFB (breakage-fusion-bridge), are separately indicated. (8) SV: Types of structural variation including deletions (DEL), inversions (INV), duplications (DUP), and translocations (TRA) are distinguished by labels. Detailed results can be interactively viewed using the CCRR web server interface.

As depicted in Fig. 1A, a complete CCRR workflow begins with whole-genome sequencing of a tumor/normal sample pair. SV detection is conducted using six SV callers—Manta, Gridss (Cameron *et al.* 2021), Lumpy (Layer *et al.* 2014), SvABA (Wala *et al.* 2018), SoReCa (Rosswog *et al.* 2021), and Delly—which analyze the input samples and merge their outputs to produce highly reliable consensus SVs. Delly also works alongside Sclust (Cun, 2018), Sequenza (Favero *et al.* 2015), PURPLE (Priestley *et al.* 2019), and CNVkit (Talevich *et al.* 2016) to generate and merge copy number states. Varscan (Koboldt *et al.* 2012) generates the somatic mutation data required by Sclust. PURPLE’s execution incorporates three recommended software: Amber, which generates BAF; Cobalt, which generates read depth ratios; and Sage, which generates somatic mutations for PURPLE (Priestley *et al.* 2019). Purity and ploidy information is inferred by PURPLE or Sequenza, with preference given to Sequenza’s results. Apart from Sclust’s sensitivity parameter alpha set to the default value of 0.15, all tools are run with default settings.

After obtaining tumor purity and ploidy along with consensus SVs and CNVs, six complex rearrangement callers—Shatterseek, CTLPScanner, SeismicAmplification (Rosswog *et al.* 2021), AmpliconSuite (Luebeck *et al.* 2024), Starfish (Bao *et al.* 2022), and gGnome (mskilab-org/gGnome: R API for browsing, analyzing, and manipulating reference-aligned genome graphs in a GenomicRanges framework. GitHub 2021)—will each operate independently. Shatterseek, Starfish, and SeismicAmplification detect complex structural variation events such as chromothripsis and seismic amplification based on SVs and CNVs. CTLPScanner requires only CNVs to detect chromothripsis patterns. AmpliconSuite facilitates the operation of AmpliconArchitect, categorizing its analysis results into three types of amplification events: BFB cycles, ecDNA, and linear amplifications, and additionally requires original BAM files. JaBba (Hadi *et al.* 2020) uses the original BAM files, consensus SVs and CNVs, purity, and ploidy to build a genome graph for gGnome. gGnome, based on JaBba's output, detects and categorizes complex rearrangement events, such as chromothripsis and chromoplexy. Finally, CCRR utilizes R packages such as circlize to summarize and visualize the analysis results (Fig. 1B). The primary tools and their functions used in the workflow are summarized in Table 1, available as supplementary data at *Bioinformatics* online.

### 2.2 Merge SVs and get consensus CN status

To generate a consensus SV, outputs from all SV callers are standardized and organized by identifying related SVs based on consistent chromosome orientation and a position difference within 150 bp. Related SVs are then aggregated, with the consensus SV defined by the common chromosome, orientation, and mode position. The confidence level is determined by the sources of entries in the aggregated collection.

Consensus CN status is derived similarly to the PCAWG approach (Dentro *et al.* 2021), combining and smoothing outputs from all CNV callers to create a comprehensive genome-wide CN status. Each CN alteration expands 5000 bp upstream and downstream, with overlapping regions divided equally. Intersecting CN regions form a consensus alteration site, and the CN status is weighted by the proportion of different CN areas. Within the consensus CN interval, each tool’s bias (deviation from consensus) and volatility (consistency) are measured and scaled for comparison.

### 2.3 CCRR web server

To facilitate usage, we have developed the CCRR web server. Due to the large size of BAM files and the associated transfer and computational burdens, the CCRR web service is designed to start from preprocessed SV and CNV result files rather than raw BAM files. AmpliconArchitect, which requires BAM files as input, is excluded from the complex rearrangement analysis process.

Users can generate SV and CNV result files from their original files using any supported SV and CNV caller locally. They can then upload these result files to the CCRR web server. By selecting the desired parameters and clicking “Start”, users can seamlessly perform the five types of complex rearrangement analyzes—Shatterseek, CTLPScanner, SeismicAmplification, Starfish, and gGnome—and view the analysis results and summaries on the results page (Fig. 1, available as supplementary data at *Bioinformatics* online). An embedded application allows users to customize the regions, styles, and colors of the CIRCOS plot.

## 3 Examples

To evaluate the performance of the CCRR workflow, we used gold standard data from a multi-center, multi-technology consensus study on tumor structural variations (Talsania *et al.* 2022). This dataset systematically examined the breast cancer cell line HCC1395/HCC1395BL using various sequencing platforms, establishing a consensus SV set validated by multiple orthogonal methods. The BAM files of different depths were processed through the CCRR workflow with default parameters (Table 2, available as supplementary data at *Bioinformatics* online). Consensus SVs identified by multiple tools demonstrated higher accuracy. For example, in sample IL_1, SVs from three or more callers were merged using default CCRR parameters (Fig. 2, available as supplementary data at *Bioinformatics* online), yielding 769 SVs, with over half supported by at least five callers. This increased accuracy from SvABA’ s 53.46%–59.3%. CNV results from five callers were also merged, with most copy number segments being under 1 Mb and none exceeding 10 Mb. As segment length increased, bias and volatility tended to converge.

Resource usage for sample IL_1 was statistically tracked throughout the process using ContainerProfiler (Hoang *et al.* 2022), with the system configured as an Intel(R) Xeon(R) Platinum 8168 CPU @ 2.70 GHz. The complete analysis of sample IL_1 on a high-performance server, limited to a maximum of 30 threads and 200GB of memory, took a total of 145.13 h, primarily running on a single thread (Fig. 3, available as supplementary data at *Bioinformatics* online). Tools such as Manta, SvABA, Gridss, PURPLE, and CNVkit support parallel computation. On a PC, sample FD_1 was tested on a personal computer equipped with an Intel(R) Core(TM) i7-11800H @ 2.30 GHz, 32GB of memory, and limited to a maximum of 4 threads and 12GB of memory. The CCRR workflow on the PC ran at speeds close to those on the high-performance server (Table 3, available as supplementary data at *Bioinformatics* online). The total processing time was about 132.6 h. For faster analysis, longer-running single-threaded modules such as Sequenza and Sclust can be optionally skipped.

## Supplementary Material

btaf386_Supplementary_Data

## Data Availability

The data underlying this article are available in the NCBI SRA database at https://www.ncbi.nlm.nih.gov/sra?term=SRP162370. The datasets were derived from sources in the public domain, including the NCBI FTP site: ftp://ftp-trace.ncbi.nlm.nih.gov/ReferenceSamples/seqc/Somatic_Mutation_WG/.
