# Supplementary material for: CCRR: a user-friendly platform for analyzing complex chromosomal rearrangements in tumors
Source: Bioinformatics. 2025 Jul 3;41(7):btaf386. doi: 10.1093/bioinformatics/btaf386 (PMC12258142; doi:10.1093/bioinformatics/btaf386)
Supplement: btaf386_Supplementary_Data [file btaf386_supplementary_data.zip › Supplementary Figure S1.pdf]

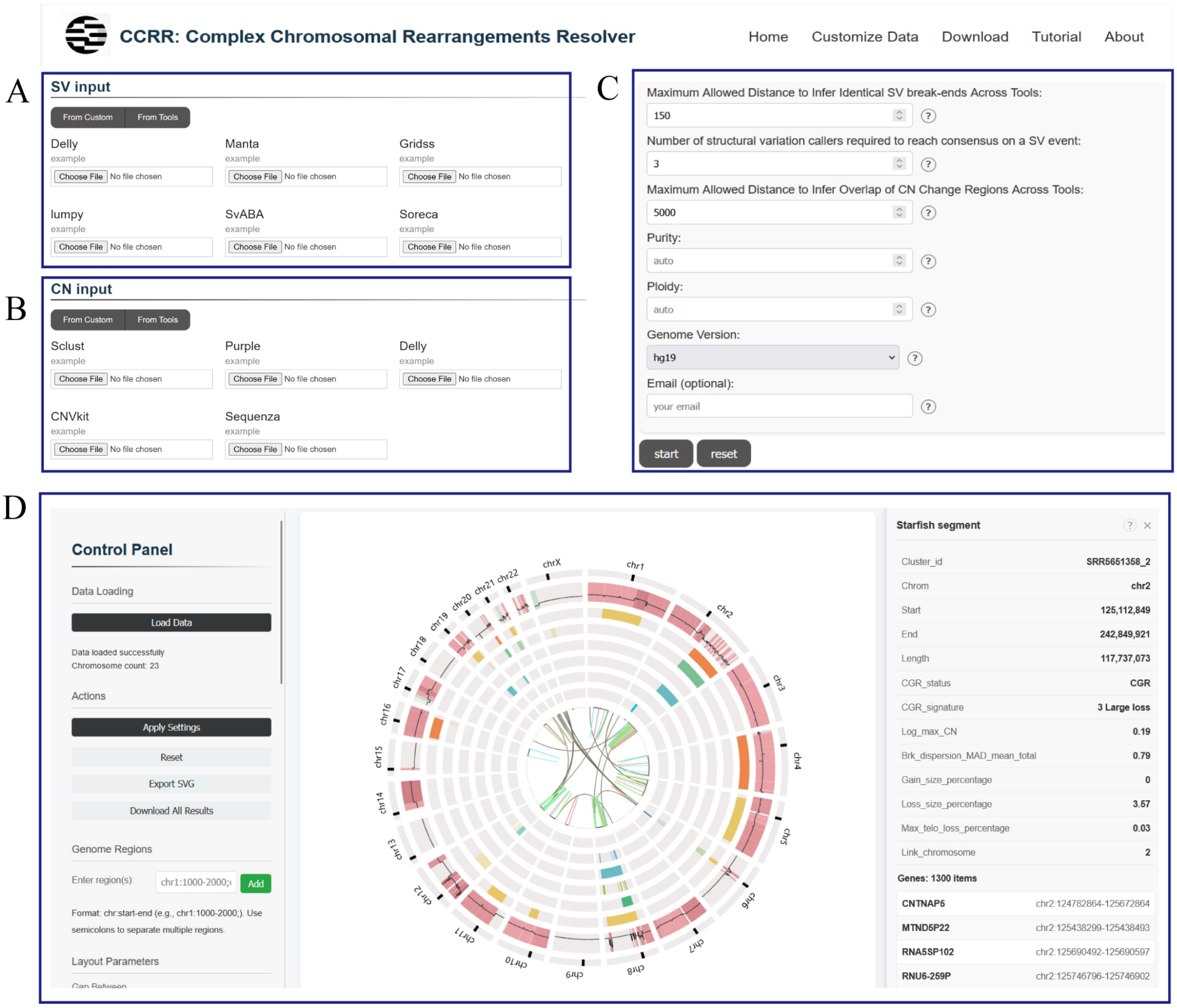

Figure S1. Usage of the CCRR Webserver. (A) Select "From tools" to upload results from various SV callers, or select "From custom" to upload custom SVs. Click on "example" to view the format of each file. (B) Select "From tools" to upload results from various CNV callers, or select "From custom" to upload custom CNVs. Click on "example" to view the format of each file. (C) Select "Options" to set the relevant parameters, or run with the default parameters. Click "Start" to begin the analysis, or click " reset" to start a new input. After several minutes to an hour, the results page will be displayed. If an email address has been provided in the options, a link to the results page will be sent to the email upon completion of the analysis. (D) Use the left control panel to load data, customize settings, or export results. Click any element in the Circos plot to view detailed annotations on the right panel.
