# Supplementary material for: CCRR: a user-friendly platform for analyzing complex chromosomal rearrangements in tumors
Source: Bioinformatics. 2025 Jul 3;41(7):btaf386. doi: 10.1093/bioinformatics/btaf386 (PMC12258142; doi:10.1093/bioinformatics/btaf386)
Supplement: btaf386_Supplementary_Data [file btaf386_supplementary_data.zip › Supplementary Figure S2.pdf]

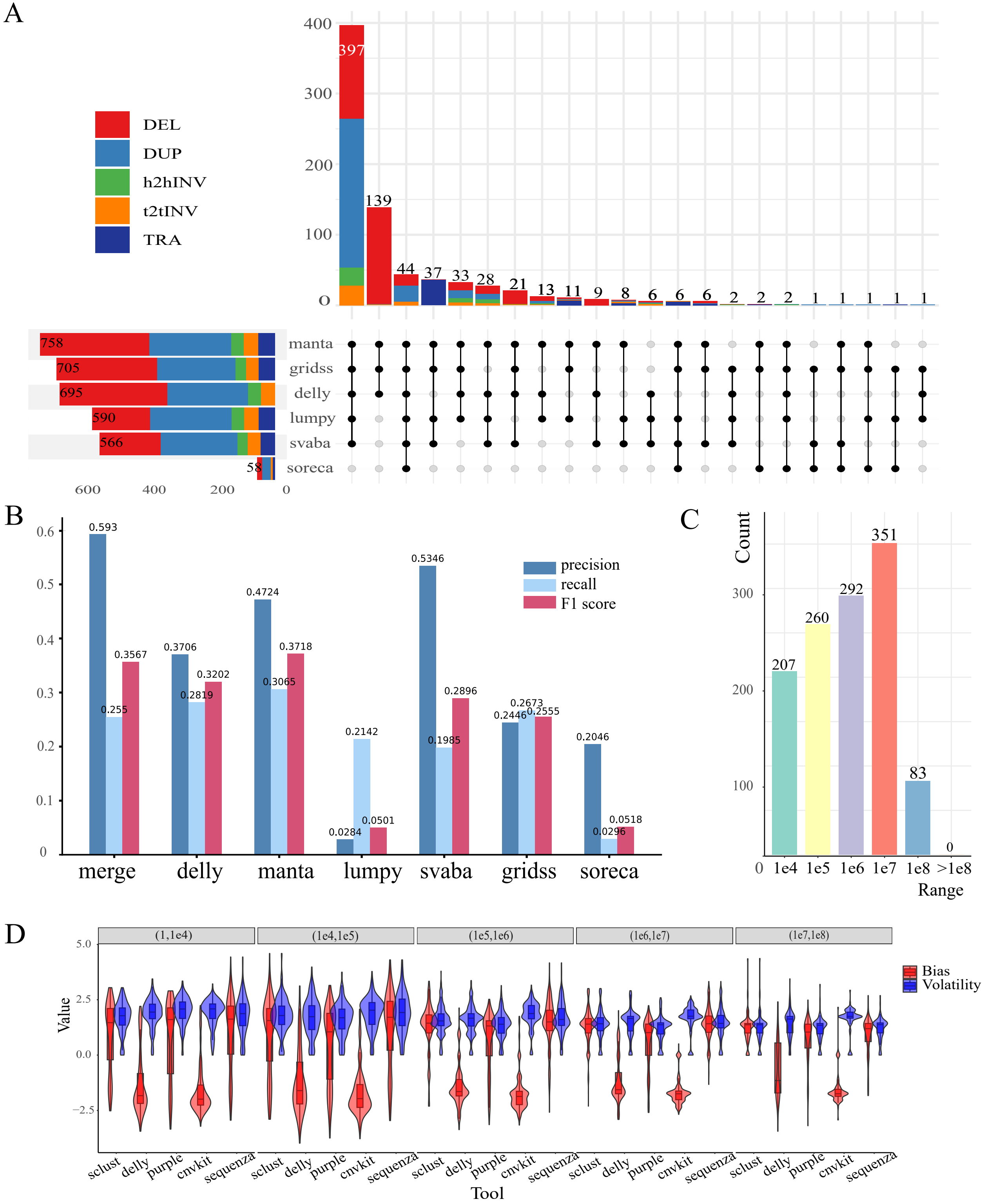

A
DEL
DUP
h2hINV
t2tINV
TRA
400
300
200
100
0
397
139
44
37
33
28
21
13
11
9
8
6
6
6
2
2
2
1
1
1
1
1
758
705
695
590
566
58
manta
gridss
delly
lumpy
svaba
soreca
600
400
200
0
B
0.6
0.5
0.4
0.3
0.2
0.1
0
precision
recall
F1 score
0.593
0.255
0.3567
0.3706
0.2819
0.3202
0.4724
0.3065
0.3718
0.0284
0.2142
0.0501
0.5346
0.1985
0.2896
0.2446
0.2673
0.2555
0.2046
0.0296
0.0518
merge
delly
manta
lumpy
svaba
gridss
soreca
C
Count
300
200
100
207
260
292
351
83
0
0
1e4
1e5
1e6
1e7
1e8
>1e8
Range
D
(1,1e4)
(1e4,1e5)
(1e5,1e6)
(1e6,1e7)
(1e7,1e8)
5.0
2.5
0.0
−2.5
Value
Bias
Volatility
sclust
delly
purple
cnvkit
sequenza
Tool

Figure S2. Overview of SV and CN Merging for Sample IL_1. (A) Distribution, sources, and consensus of all merged SVs. (B) Precision, recall, and F1 scores of the merged SVs and the six SV callers compared to the gold standard. (C) Length distribution of the merged copy number segments. (D) Bias and volatility of each tool for consensus copy numbers within different segment length ranges.
