# Supplementary material for: CCRR: a user-friendly platform for analyzing complex chromosomal rearrangements in tumors
Source: Bioinformatics. 2025 Jul 3;41(7):btaf386. doi: 10.1093/bioinformatics/btaf386 (PMC12258142; doi:10.1093/bioinformatics/btaf386)
Supplement: btaf386_Supplementary_Data [file btaf386_supplementary_data.zip › Supplementary Table S1.docx]

**Table S1 A summary of the primary tools, algorithms, and R packages utilized in the CCRR workflow**

| Tool | Version | Function |
| --- | --- | --- |
| Manta | 1.6.0 | Detect SV |
| Gridss | 2.13.2 | Detect SV |
| Lumpy | 0.3.1 | Detect SV |
| SvABA | 1.1.0 | Detect SV |
| SoReCa | 0.6.2 | Detect SV |
| Varscan | 2.4.6 | Generate somatic mutations for Sclust |
| Sclust | - | Detect CNV |
| Sequenza | 3.0.0 | Infer purity and ploidy , detect CNV |
| Amber | 3.9 | Generate BAF for PURPLE |
| Cobalt | 1.13 | Generate read depth ratios for PURPLE |
| Sage | 3.2.3 | Generate somatic mutations for PURPLE |
| PURPLE | 3.7.1 | Infer purity and ploidy , detect CNV |
| CNVkit | 0.9.10 | Detect CNV |
| Shatterseek | 1.1 | Detect chromothripsis |
| CTLPScanner | 1.0.0 | Detect Chromothripsis-like patterns |
| SeismicAmplification | - | Detect Seismic Amplification |
| AmpliconSuit | 1.3.2 | Detect ecDNA |
| Starfish | 0.11 | Detect Complex genomic rearrangements (CGRs) signatures |
| JaBba | 1.1 | Build a genome graph for gGnome |
| gGnome | 1.0 | Detect multiple rearrangement events |
