# Supplementary material for: CCRR: a user-friendly platform for analyzing complex chromosomal rearrangements in tumors
Source: Bioinformatics. 2025 Jul 3;41(7):btaf386. doi: 10.1093/bioinformatics/btaf386 (PMC12258142; doi:10.1093/bioinformatics/btaf386)
Supplement: btaf386_Supplementary_Data [file btaf386_supplementary_data.zip › Supplementary Table S2.docx]

**Table S2 Information on Benchmark Samples**

| File | Reference | Average depth | Size |
| --- | --- | --- | --- |
| WGS_FD_N_1.bam/WGS_FD_T_1.bam | GRCh38 | 42/40 | 61G/63G |
| WGS_EA_N_1.bam/WGS_EA_T_1.bam | GRCh38 | 60/63 | 95G/107G |
| WGS_IL_N_1.bam/WGS_IL_T_1.bam | GRCh38 | 60/68 | 114G/128G |
