# Supplementary material for: CCRR: a user-friendly platform for analyzing complex chromosomal rearrangements in tumors
Source: Bioinformatics. 2025 Jul 3;41(7):btaf386. doi: 10.1093/bioinformatics/btaf386 (PMC12258142; doi:10.1093/bioinformatics/btaf386)
Supplement: btaf386_Supplementary_Data [file btaf386_supplementary_data.zip › Supplementary Table S3.docx]

**Table S3 Analysis times for each module of the CCRR workflow on a PC for FD_1**

| Tool or module | Time | Tool or module | Time |
| --- | --- | --- | --- |
| Sequenza | 18.5h | Sclust | 50.1h |
| Delly | 13.9h | Gridss | 4.5h |
| Manta | 3.0h | PURPLE | 5.2h |
| SoReCa | 9.2h | CNVkit | 7.9h |
| Lumpy | 11.6h | Merge | 0.3h |
| SvABA | 6.1h | Complex Analysis | 2.5h |
