# Supplementary figures and images for: CCRR: a user-friendly platform for analyzing complex chromosomal rearrangements in tumors
Source: Bioinformatics. 2025 Jul 3;41(7):btaf386. doi: 10.1093/bioinformatics/btaf386 (PMC12258142; doi:10.1093/bioinformatics/btaf386)

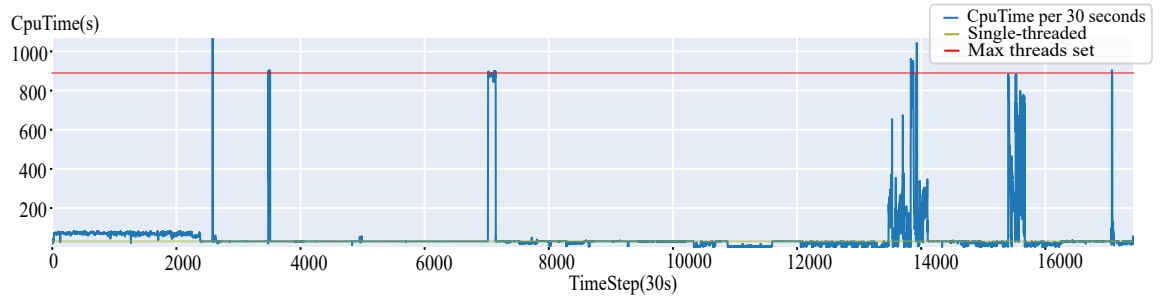

Supplement: btaf386_Supplementary_Data [file btaf386_supplementary_data.zip › Supplementary Figure S3.pdf]
